# Supplementary material for: Gelatin/Chitosan Bilayer Patches Loaded with Cortex Phellodendron amurense/Centella asiatica Extracts for Anti-Acne Application
Source: Polymers (Basel). 2021 Feb 15;13(4):579. doi: 10.3390/polym13040579 (PMC7918957; doi:10.3390/polym13040579)
Supplement: Supplementary file 1 [file polymers-13-00579-s001.pdf]

Supplementary Materials

Fig S1.

A

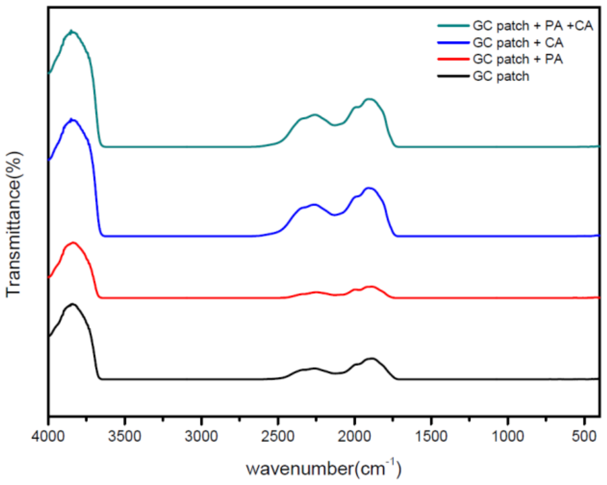

B.

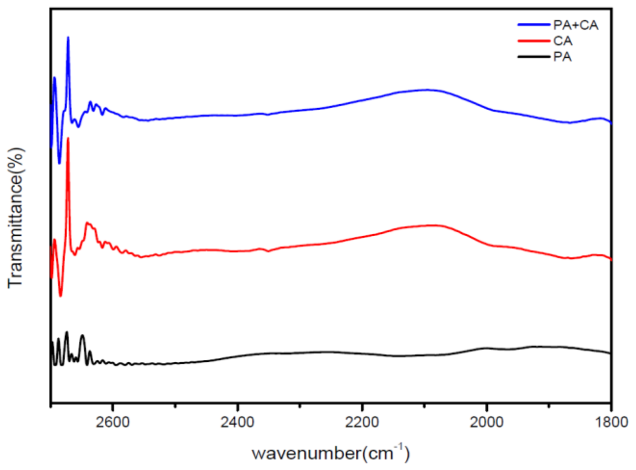

Figure S1

(A) FTIR analysis of 4 types of bilayer patches (GC, GC/PA, GC/CA, and GC/ PA+CA). (B) FTIR analysis of the addition of herbal extracts (PA, CA, and PA+CA). GC patch is used as the control group
